# Antigen-specific Treg cells in immunological tolerance: implications for allergic diseases

**DOI:** 10.12688/f1000research.12650.1

**Published:** 2018-01-10

**Authors:** Azza Abdel-Gadir, Amir H. Massoud, Talal A. Chatila

**Affiliations:** 1 Division of Immunology, Boston Children’s Hospital, Boston, USA; 2 Department of Pediatrics, Harvard Medical School, Boston, USA

**Keywords:** Regulatory T Cells, allergy, antigen-specific

## Abstract

Allergic diseases are chronic inflammatory disorders in which there is failure to mount effective tolerogenic immune responses to inciting allergens. The alarming rise in the prevalence of allergic diseases in recent decades has spurred investigations to elucidate the mechanisms of breakdown in tolerance in these disorders and means of restoring it. Tolerance to allergens is critically dependent on the generation of allergen-specific regulatory T (Treg) cells, which mediate a state of sustained non-responsiveness to the offending allergen. In this review, we summarize recent advances in our understanding of mechanisms governing the generation and function of allergen-specific Treg cells and their subversion in allergic diseases. We will also outline approaches to harness allergen-specific Treg cell responses to restore tolerance in these disorders.

## Introduction

Regulatory T (Treg) cells play a key role in the maintenance of immunological self-tolerance and in restraining deleterious immune responses to both self and foreign antigens ^1^. Treg cell–mediated tolerance is an active process that requires antigen specificity ^2, 3^ and this dependence on antigen recognition by Treg cells for their regulatory function can be harnessed to provide promising approaches for immunotherapy of diseases such as allergy and autoimmunity.

Sakaguchi *et al*. originally described a population of CD4 ^+^ T cells expressing the cell surface marker CD25—interleukin-2 (IL-2) receptor alpha chain—that maintained peripheral immunological tolerance in mice. The deficiency of these cells engenders autoimmunity ^4^. The discovery of a key role for the transcription factor Forkhead Box P3 (FOXP3) in controlling the differentiation and functions of CD4 ^+^CD25 ^+^ Treg cells provided an essential molecular framework for elucidating their physiological functions ^5^. It is now appreciated that Treg cells have a broader function than previously thought. In addition to maintaining tolerance to self-tissues, Treg cells are implicated in sustaining tolerance to fetal and transplanted tissues and in promoting tissue repair ^6– 9^. Furthermore, Treg cells promote tolerance to components of the ‘extended self’, encompassing the commensal microbiota and innocuous agents such as nutrients and other environmental exposures ^10, 11^. Although additional Foxp3 ^–^ Treg cell populations have been described ^12^, this review is focused on CD4 ^+^CD25 ^+^Foxp3 ^+^ Treg cells, the most well-characterized subset of Treg cells whose deficiency in both humans and mouse models precipitates lethal autoimmunity and inflammatory disorders.

## What are the niches that promote antigen-specific regulatory T cells formation?

**Figure 1.**
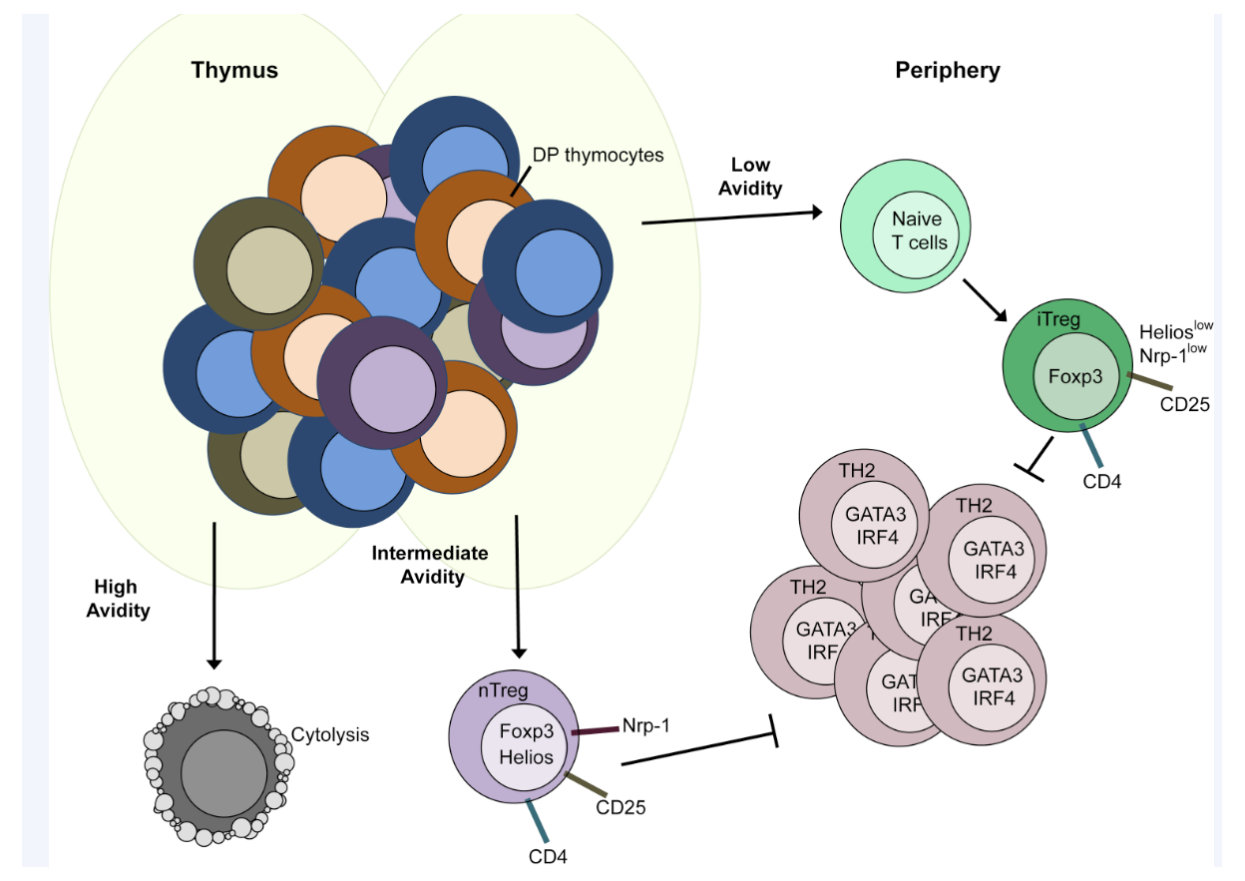
Generation of natural regulatory T (nTreg) and induced Treg (iTreg) cells. Schematic representation of nTreg and iTreg cell developmental pathways. Naturally occurring Treg cells develop upon intermediate-avidity interaction between developing thymocytes and self-antigen-presenting medullary epithelial cells. High-level expression of Neuropilin (Nrp-1) and Helios is maintained on nTreg cells after their migration from the thymus. Naïve conventional T cells that have undergone positive and negative selection in the thymus and have low avidity for self-antigens may develop into iTreg cells upon encountering antigens presented by tolerance-inducing antigen-presenting cells in specialized niches in the periphery. These cells express low levels of Helios or Nrp-1. Both nTreg and peripheral Treg cells are able to suppress CD4 ^+^ effector helper T 2 (TH2) cell responses. High-avidity T-cell receptor interaction in the thymus results in cytolytic T cells (negative selection). DP, double positive; IRF4, interferon gamma regulatory factor 4.

There are two major subtypes of CD4 ^+^Foxp3 ^+^ Treg cells ^13^. The first encompasses thymic or natural Treg (nTreg) cells, which originate as a separate cell lineage during T-cell development in the thymus ( Figure 1) ^14, 15^. The second type includes peripheral or induced Treg (iTreg) cells that differentiate from conventional T (Tconv) cells in specialized extra-thymic niches ^16– 20^ (reviewed in 18). nTreg cells are biased toward immune recognition of self-antigens, whereas iTreg cells are biased toward recognizing non-self-antigens, reflecting their derivation from Tconv cells ^18, 21^. The two subtypes are complementary in their actions in promoting peripheral tolerance ^22– 24^.

nTreg cells emerge late in the double-positive stage of thymocyte development ^25^, and the majority of nTreg cells segregate into CD4 single-positive mature thymocytes ^21^. The maturation of nTreg cells from thymocytes requires T-cell receptor (TCR)-dependent signals delivered upon its engagement by major histocompatibility complex (MHC) II molecules on specialized antigen-presenting cells ^26^, most notably medullary thymic epithelial cells ^27, 28^. Whereas high-avidity TCR/MHC interactions induce negative selection ^29, 30^ and low-avidity interactions favor thymocyte maturation and egress as conventional naïve T cells, intermediate-avidity interactions favor FOXP3 expression and the differentiation of thymocytes into nTreg cells ( Figure 1) ^31– 33^. The TCR repertoire expressed by nTreg cells is broad and distinct from Tconv cells. It is enriched in self-reactive TCRs that otherwise would have been eliminated in Tconv cells ^34, 35^.

**Figure 2.**
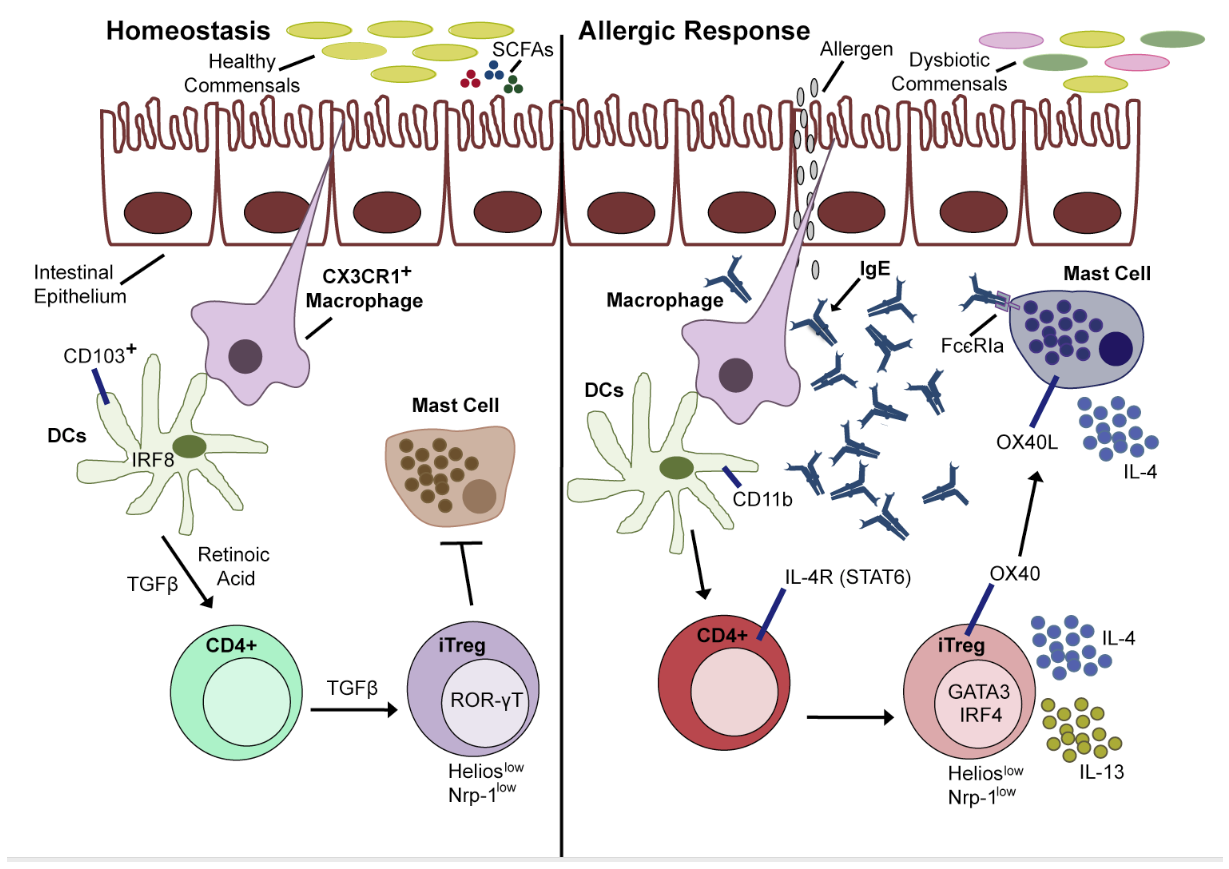
Mucosal tolerance in the gut and its breakdown in food allergy. ( **Left**) Under homeostatic conditions, CX3CR1 ^+^ macrophages sample components of the gut lumen and transfer luminal antigens to IRF8 ^+^CD103 ^+^ dendritic cells (DCs), which in turn promote the formation of antigen-specific induced regulatory T (iTreg) cells. iTreg cells expressing retinoid-related orphan receptor gamma t (RoR-γt) may regulate tolerance to dietary antigens by a range of mechanisms, including the suppression of mast cell activation. iTreg cells may also prevent the conversion of naïve CD4 ^+^ T cells to antigen-specific helper T 2 (TH2) cells. ( **Right**) Under food allergic conditions, CD11b ^+^CD103 ^–^ DCs encounter allergens that are made more accessible by a permeable epithelial barrier. Allergen-presenting DCs drive the differentiation of naïve T cells into allergen-specific TH2 cells expressing the interleukin-4 receptor (IL-4R). iTreg cells also acquire a TH2 cell-like phenotype characterized by increased GATA3 and IRF4 expression and the release of IL-4 and IL-13. These ‘pathogenic’ Treg cells are unable to suppress mast cell activation through engagement of the OX40-OX40 ligand pathway. Binding of IgE to the high-affinity receptor for IgE (FcεRI) leads to uninhibited mast cell degranulation and IL-4 production. IRF8, interferon gamma regulatory factor 8; SCFA, short-chain fatty acid; TGFβ, transforming growth factor beta.

iTreg cells are generated in specialized niches particularly at the environmental interfaces, including the gut and lung ^20, 36^. These niches are endowed with specialized antigen-presenting cells—including CD103 ^+^CD11c ^+^ dendritic cells in the gut and alveolar and interstitial macrophages in the lung—that polarize antigen-responsive T cells into iTreg cells under the influence of transforming growth factor beta 1 (TGF-β1) and retinoic acid produced by the antigen-presenting cells ( Figure 2) ^37– 41^. In the intestine, iTreg cells develop primarily in response to the gut microbiota and food antigens, enabling tolerance to both sets of foreign antigens ^42– 44^. In germ-free (GF) mice, colonic Treg cells generated in the periphery (iTreg cells) are greatly reduced in numbers, reflecting the critical role of colonic bacteria in promoting colonic Treg cell development ^45^. In contrast, GF mice appear to have normal numbers of iTreg cells in the small intestine ^46^. Kim *et al*. reported that under normal physiological conditions, proteins from a typical diet lead to iTreg cell generation in the small intestine but not in the colon ^11^. They found that phenotypically and functionally distinct iTreg cells in the lamina propria of the small intestine develop after weaning to suppress strong immunological reactions in response to dietary antigens in solid food. These iTreg cells are important for the maintenance of immune tolerance at mucosal surfaces. Compared with nTreg cells, they express lower levels of the markers Neuropilin-1 and Helios and higher levels of the retinoid-related orphan receptor gamma t (RoR-γT) ^11^.

The lung is another important site for the induction of antigen-specific Treg cells ^20, 47, 48^. Although alveolar macrophages are defective in their capacity to activate T effector (Teff) cells, they are particularly adept at driving the differentiation of naïve allergen-specific T cells into iTreg cells in a TGF-β1- and retinoic-dependent manner ^37, 38^. Similar to that of the gut, the microbiota of the lung has been implicated in promoting airway tolerance to allergens early in life by enabling the production of allergen-specific induced Treg cells ^49^. High frequencies of Treg cells targeting innocuous environmental aero-antigens (such as house dust mite or plant pollen) have been reported in human adult peripheral blood mononuclear cells but are absent in cord blood, suggesting that Treg cell expansion is specific to antigen encounter in the periphery ^50^. Aero-antigen-targeted responses were also found to be less pronounced in Tconv cells as Treg cells outnumbered effector memory T (TM) cells and expressed chemokine receptors essential for lung homing ^50^.

Feedback mechanisms from the peripheral niches in the form of local tissue factors have been shown to regulate the function of Treg cells. In the skin, the pro-inflammatory cytokine thymic stromal lymphopoietin (TSLP) produced by keratinocytes acts to activate Treg cells to contain skin inflammation locally and prevent its systemic spread ^51^. Deletion of TSLP receptor (TSLPR) signaling specifically on Treg cells prevented the upregulation of genes, including *Icos* and *Ctla4*, that mediate their regulatory effects and consequently restricted the capacity of Treg cells to inhibit skin effector T cells and prevent the progression of local skin inflammation systemically.

## Treg cell memory

One of the key features of adaptive immunity is the ability to respond effectively and more rapidly to antigens that have been encountered previously. A subset of clonally expanded Teff cells differentiate into long-lived TM cells that are particularly adept at mounting recall responses upon re-exposure to antigens/pathogens ^52^. TM cells are endowed with long-term epigenetic, transcriptional, and metabolic changes that enable their long-term survival and rapid response to recall challenges ^53^. The concept of Treg cell memory has been controversial as a long-lasting increase in suppressive function that persists after the resolution of the primary antigenic exposure could severely suppress recall Teff cell responses and lead to persisting immuno-suppression ^54– 57^. Van der Veeken *et al*. recently found that the suppressive function of antigen-specific Treg cells is a transient quality ^58^. They reported that inflammation-induced Treg cells reversed activation-specific transcriptional changes and reduced suppressive function with time. These changes were associated with stable chromatin modifications that facilitate reactivation and long-lasting preference for non-lymphoid tissue localization. Rested Treg cells exhibited a gene expression signature similar to that of conventional TM cells that was not affected by secondary activation ^58^.

In contrast to the above results, other studies on Treg cell responses have supported the concept of Treg cell memory. Rosenblum *et al*. investigated Treg cell responses in the skin by generating transgenic mice that expressed a model antigen in keratinocytes ^59^. The model antigen was regulated in the skin but only constitutively expressed in the thymus and the expression could be silenced to assess antigen-specific TM cells without prolonged exposure to antigen. This system revealed that the thymic constitutive expression allowed the expansion of a large subset of antigen-specific Treg cells that spread to secondary lymphoid organs. The induction of antigen in the skin induced potent proliferation of antigen-specific Treg cells and increased expression of CTLA-4 before migrating to the skin to ameliorate antigen-specific effector T-cell inflammation. After antigen expression was switched off, a subset of Treg cells expressing CTLA-4 persisted and upon antigen re-exposure inflammation was attenuated much more rapidly compared with the primary response ^59^. Treg cell depletion between primary and secondary exposure saw no protection from inflammation. Overall, these results are relevant to therapeutic applications aimed at boosting tolerance responses, such as immunotherapy for allergic diseases (see below).

## What is the role of antigen-specific Treg cells in allergic diseases?

Allergic responses arise in the context of failure to develop tolerance toward specific allergens. These conditions are marked by the production of allergen-specific IgE and the development of CD4 ^+^ TH2 skewed cell responses ( Figure 2) ^60^. Conditions that impact the differentiation of allergen-specific Treg cells can give rise to allergic disorders ^60^. For example, human subjects with loss-of-function mutations in FOXP3, whose Treg cells fail to appropriately differentiate into effective suppressors, develop an X-linked disorder of immune dysregulation and autoimmunity ^61– 63^. A similar phenotype is recapitulated in mice with mutations in Foxp3 ^64– 67^. Human subjects with FOXP3 deficiency suffer from associated severe allergic inflammation and develop food allergy (FA) due to loss of oral tolerance. In mice, manipulations that deplete or inhibit allergen-specific Treg cells also result in the loss of tolerance and the development of allergic responses to said allergen ^68^. In human subjects, Treg cells of children with FA fail to suppress responses to the causative food allergens, whereas those of children who outgrow FA develop allergen-specific suppressive capacity ^68^.

## Derangement of antigen-specific Treg cell responses in allergic diseases

Oral tolerance is specifically associated with the development of iTreg cells from naïve CD4 ^+^ T cells that are activated in the presence of TGF-β1 and CD103 ^+^ classic dendritic cells in the gut that express interferon gamma regulatory factor 8 (IRF8) ( Figure 2) ^41, 69^. At steady state, macrophages expressing the CX3C chemokine receptor 1 (CX3CR1) sample luminal antigens, including food and microbiota components, by projecting dendrites through the epithelial barrier into the gut lumen ^70– 72^. CX3CR1 ^+^ macrophages transfer the luminal soluble antigen to CD103 ^+^ dendritic cells, which in turn induce oral tolerance to said antigens by promoting the generation of antigen-specific iTreg cells ( Figure 2) ^73^. iTreg cells regulate TH2 immune responses at mucosal surfaces, are less stable, and exhibit more plasticity than thymic-derived nTreg cells ^74, 75^. Noval Rivas *et al*. showed that, in a mouse model of FA marked by IgE-mediated anaphylaxis, the generation of antigen-specific Treg cells was inhibited by increased IL-4 receptor (IL-4R)-STAT6 signaling ^68^. The mice in question, *Il4ra* ^F709^ mice, carry a gain-of-function mutation in the IL-4Rα chain ^76^. They mount exaggerated TH2 responses and are particularly susceptible to oral sensitization and anaphylaxis ^77^. The antigen-specific Treg cells that did develop underwent TH2 cell reprogramming marked by increased expression of IL-4, IL-13, IRF-4, and GATA-3 when compared with those from wild-type (WT) mice, a phenomenon also observed in human subjects with FA ( Figure 2). The impaired induction of food allergen-specific iTreg cells paralleled the excessive IL-4R signaling and could be reversed by the deletion of Stat6.

GATA-3 plays a key role in Treg cell homeostasis, acting to prevent polarization to TH17 cells ^78, 79^. Under physiological conditions, TH cell reprogramming is restrained and is lost in the presence of increased STAT6 signaling, thereby contributing to the pathogenic reprogramming of Treg cells into TH2-like cells ^68^. Treg cell lineage-specific deletion of *Il4* and *Il13* genes abrogates the induction of FA, thus confirming that IL-4 production by TH2 cell-like reprogrammed Treg cells directly contributes to allergic disease ^68^. The *in vitro* suppression of mast cell activation by antigen-specific Treg cells was abrogated in the presence of IL-4 but reversed with the deletion of *Stat6* in Treg cells. This is supported by previous reports that the suppression of mast cell activation and IL-4 production restores tolerance and promotes the induction of Treg cells ^80^. Although the programming of iTreg cells into TH2 cell-like cells is pathogenic in FA, it may serve physiological purposes under other circumstances. For example, intense IL-4/IL-4R signaling in the context of helminth infections has been reported to drive the development of TH2 cell-like ex-Treg cells, which contribute to immunity to nematodes ^81^.

The above concepts of iTreg cell suppression and pathogenic reprogramming into Teff-like cells, developed in the context of FA, have been extended to encompass the pathogenesis of other allergic diseases such as asthma. The frequencies of suppressive allergen-specific Treg cells trend higher in healthy controls as compared with asthmatics ^82^. Importantly, there is evidence of pathogenic reprogramming of Treg cells toward effector phenotypes that contribute to asthma severity ^83^. Infection with respiratory syncytial virus induced a TH2 cell-like effector program in Treg cells and impaired their suppressive function ^84^. Also, TH2 cell-like reprogramming of iTreg cells due to enhanced STAT6 activation via the IL-4Rα in *Il4ra* ^F709^ mice is associated with intense TH2 cell-skewed allergic airway inflammation. Other Treg cell pathways have also been implicated in the regulation of the TH2 cell response in allergic airway inflammation. Deletion of the beta subunit of casein kinase 2 (CK2) resulted in the proliferation of a Treg cell subpopulation characterized by the expression of the inhibitory receptor ILT3 (also known as gp49B). This Treg cell population was unable to control the maturation of dendritic cells expressing both the transcription factor IRF4 and the programmed cell death ligand 2 (PD-L2), which drove the development of lung TH2 responses ^85^.

**Figure 3.**
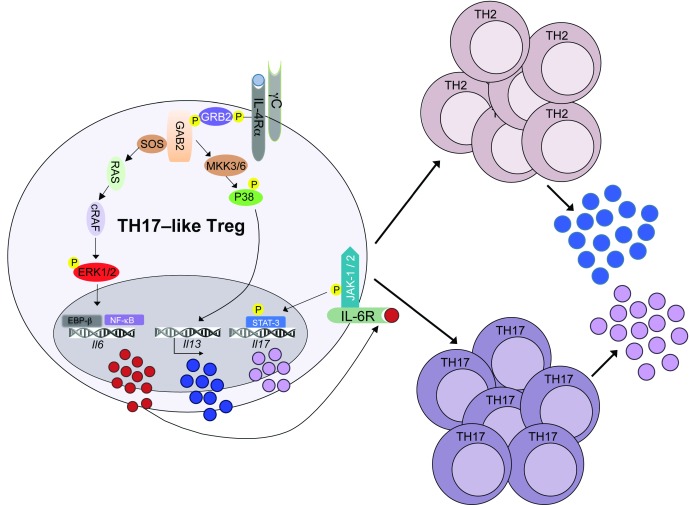
Induced regulatory T (iTreg) cell subversion by the asthma-promoting IL-4Rα-R576 variant. Schematic representation of pathways mediating iTreg cell subversion by the asthma-promoting IL-4Rα-Q76R variant. The glutamine to arginine substitution at position 576 of IL-4Rα chain allows recruitment of the protein adaptor GRB2 upon receptor activation. GRB2 activates downstream MAP kinase cascades, including extracellular signal–regulated kinases, which drive IL-6 production by activating the transcription factors NF-κB and C/EBP-β, and p38 MAP kinase, which activates IL-13 production. Newly formed antigen-specific iTreg cells are subsequently destabilized, resulting in the generation of excessive TH2 and TH17 cell responses that promote severe airway hyper-responsiveness.

More recently, we described a novel mechanism involving a common asthma-promoting human IL-4Rα chain variant, by which allergen-specific iTreg cell differentiation is subverted to promote mixed TH2/TH17 cell inflammation, associated with severe, steroid-resistant asthma ^86^. When expressed in mice, this variant, which contains a glutamine-to-arginine (Q-to-R) substitution at position 576 of the IL-4Rα, promotes exaggerated airway hyper-responsiveness and severe mixed TH2/TH17 inflammation when the mice are sensitized with allergens and subsequently challenged. The R substitution does not impact IL-4R signaling via STAT6. Rather, IL-4 signaling via IL-4Ra-R576 activates IL-6 production by inducing the *de novo* recruitment of the adaptor growth factor receptor-bound protein 2 (GRB2) to the IL-4Rα ^86^ ( Figure 3). GRB2 activates downstream MAP kinase cascades, including extracellular signal-regulated kinases to induce *IL6* gene expression by activating the transcription factors nuclear factor-kappa B (NF-κB) and C/EBP-β and p38 MAP kinase, which activates IL-13 production. Newly formed antigen-specific iTreg cells are subsequently destabilized by the confluence of IL-6 and TGF-β1 signaling, resulting in the degeneration of iTreg cells into TH17 cells that lack suppressive function. This derangement results in the over-production of both TH2 and TH17 cell responses, promoting severe airway hyper-responsiveness and inflammation. Exaggerated allergic inflammation in *Il4ra* ^R576^ mice was reversed when Treg cells differentiating into TH17 cells were inhibited by either treatment with an anti-IL-6 antibody or Treg cell-specific deletion of genes that regulate TH17 cell differentiation, including *Rorc* (encoding the TH17 master transcription factor RoR-γt) and *Il6ra*, encoding the IL-6Rα chain ^86^.

Downstream abnormalities in allergen-specific Treg cells involving immunomodulatory cytokines, including IL-10 and TGF-β, may also contribute to the pathogenesis of allergic diseases. Rubtsov *et al*. ** deleted IL-10 in Treg cells and showed increased severity of allergic airway inflammation suggesting that IL-10 production by Treg cells is critical for the induction of immune tolerance ^87^. TGF-β production by Treg cells also contributes to the regulation of the immune response ^88^. The role of altered Treg cell production of IL-10 and TGF-β in the pathogenesis of allergic diseases and the underlying mechanisms for such alterations remain to be fully elucidated.

## Antigenic specificity of allergen-specific Treg cells

The possession by nTreg cells of a distinct TCR repertoire, confirmed by several studies ^22, 34, 89, 90^, suggests that they may recognize a distinct set of peptide antigens as compared with Tconv cells ^91^. Furthermore, nTreg and iTreg cells exhibit distinct TCR repertoires, which may broaden the scope of antigens recognized collectively by the two Treg cell populations underlying their synergistic function in maintaining peripheral tolerance ^22, 92^. More recently, evidence was presented that TCR of iTreg cells may recognize peptide-MHC class II complexes with a reversed polarity as compared with the TCR of Tconv cells, again suggesting the potential for altered recognition of a distinct set of peptide antigens as compared with TCR of Tconv cells ^93^. The allergen specificity of Treg cells in humans has recently been mapped by simultaneously quantifying and characterizing allergen-reactive enriched T cells. Using this approach, Bacher *et al*. identified a population of peripherally expanded, stable Treg cells specific to innocuous aero-antigens in all allergic subjects ^50^. This subset of Treg cells served to maintain active tolerance as there was no corresponding clonally expanded high avidity-selected Tconv cell response in healthy controls. Whereas the authors did not address the suppressive influence of these antigen-specific Treg cells on TH2 induction in humans, previous mouse studies support this function.

## Allergen-specific Treg cells and the microbiome

Substantial increases in the prevalence of allergic/atopic disease have been attributed to environmental factors coupled with genetic susceptibility. The hygiene hypothesis states that recent increases in the prevalence of allergic diseases are caused by reduced microbial exposure early in age due to the use of antibiotics and improved hygiene (more sterility) ^94– 96^. Microbial colonization of neonates is initiated at birth, and the microbiotic composition of vaginally delivered infants is similar to that of the mother’s vagina. However, infants born by caesarean section obtain their microbiota from maternal skin and have been shown to have higher incidences of asthma and allergy ^97– 99^. The direct role of the microbiota in the development of allergen-specific responses is evident in GF mice that cannot be tolerized to oral antigens ^100^. We recently demonstrated that the development of FA is correlated with dysbiosis in a mouse model of allergic dysregulation ^101^. Food allergic *Il4ra* ^F709^ mice exhibited reduced abundance in members of the Firmicutes phylum and an increase in Proteobacteria phylum as compared with WT mice. The transfer of allergen-specific Treg cells blocked the development of allergic responses in mice while also preventing the food allergic dysbiosis observed in control mice ^101^.

Although the mechanisms by which the microbiota direct the differentiation and function of allergen-specific Treg cells remain to be fully elucidated, progress has been made in identifying potential pathways involved. Commensal bacteria induce the expression in iTreg cells of the transcription factor RoR-γt ^102, 103^. Ohnmacht *et al*. proposed that RoR-γt iTreg cells may regulate allergic diseases by restraining TH2 cell-mediated responses in the gut by a CTLA-4-dependent mechanism ^102^. Other studies have implicated short-chain fatty acids, including acetate, propionate, and butyrate, produced by commensals such as *Clostridial* species in stabilizing Treg cells in the gut ^104– 106^. Other microbiotic products could also be directly influencing iTreg cell differentiation and function in the gut. *Bacteroides fragilis* is a commensal bacteria that has been found to promote the upregulation of Foxp3 ^+^ Treg cells using its product, polysaccharide A (PSA), to signal through Toll-like receptor 2 in T cells ^107– 109^. *B. fragilis* lacking PSA was unable to maintain tolerance induction and upregulated TH17 cell differentiation ^110^. Failure of MyD88-dependent signaling in Treg cells severely restricted the evolution of antigen-specific Treg cell responses in the gut, consistent with the action of microbiotic products through innate immune signaling mechanisms in Treg cells promoting their expansion and function ^111^. The loss of these and other mechanisms through dysbiosis may compromise the development of Treg cells in FA and other gut dysbiotic disorders.

## Resetting pro-inflammatory antigen-specific Treg cells to promote tolerance in human subjects

The plasticity of Treg cells becomes a critically relevant issue when contemplating interventions aiming to employ Treg cells in cellular therapies or to promote Treg cell function in chronic inflammatory and autoimmune disorders. Because of the instability of Foxp3 expression in Treg cells, especially iTreg cells, under intense inflammatory conditions, these cells may acquire Teff cell phenotypes and express TH cytokines relevant to the ongoing immune response ^112^. *In extremis*, they may lose their Foxp3 expression to become pathogenic Teff cell-like ex-Treg cells ^113^. In that regard, therapies that neutralize key inflammatory cytokines have been shown to promote Treg cell stability and function under inflammatory conditions. For example, in experimental models of chronic inflammatory disorders, including arthritis and asthma, treatment with anti-IL-6 or anti-tumor necrosis factor (anti-TNF) monoclonal antibodies appears to enhance Treg cell stability and function ^114, 115^. Genetic manipulations of iTreg cells aimed to improve their stability when employed in cellular therapies may prove useful in enabling effective use of these therapies in inflammatory disorders. Conversely, therapies that are currently used in other indications to block Treg cell function such as anti-CTLA-4 and anti-PD-1 antibodies in cancer ^116^ may predispose patients to allergic diseases by inhibiting and destabilizing Treg cells, especially iTreg cells.

The induction of antigen-specific Treg cells is particularly relevant to allergen-specific immunotherapy, which aims to reduce the allergic symptoms and maintain a long-term tolerance to allergen exposure ^117^. It typically consists of gradual introduction of escalating doses of the offending allergen, leading to a state of allergen desensitization and ultimately, when successful, to a state of long-term tolerance that persists upon discontinuation of immunotherapy. A case in point is oral immunotherapy (OIT) for FA, which has the potential to modify disease outcome and, in some cases, provide a long-term cure. OIT may impact the responses of allergen-specific T cells by (a) reversing the TH2 phenotype of Teff cells or inducing their deletion or both and (b) upregulating the effectiveness of allergen-specific Treg cells ^118^. Foxp3 Treg cells and induced type 1 Treg cells have both been implicated in clinical effectiveness of OIT ^119^. For example, a role for peanut-specific Treg cells has been demonstrated in peanut-allergic patients successfully treated with OIT. Syed *et al*. reported an increase in antigen-specific Treg cells in patients who reacted well to treatment compared with those who failed to tolerize ^120^. These Treg cells were characterized with increased suppressive behavior and epigenetic modifications within the Foxp3 locus. Similarly, we observed an initial decline in Treg cells when first treated with OIT supplemented with anti-IgE therapy that were recovered over time as the patients were desensitized (Abdel-Gadir *et al*., manuscript submitted) ^121, 122^. Recovered Treg cells had increased suppressive function and reduced production of IL-4. Blockade of IL-4R signaling appeared to rescue the suppressive function of peanut-specific Treg cells isolated from untreated patients (Abdel-Gadir and Chatila, manuscript submitted). These findings suggest a possible role for IL-4R signaling blockade as an adjunct therapy in promoting long-term tolerance in OIT.

Notwithstanding these encouraging results, OIT has had varying success in conferring long-term tolerance—defined as tolerance to the ‘offending’ food for up to six months after cessation of daily OIT—in patients with FA ^123^. Moran *et al*. ^121^ and Sicherer ^122^ both report that long-term tolerance is achieved in only 13 to 28% of OIT-treated patients. The reasons for these limited long-term successes remain obscure but may have to do with the persistence of a pro-TH2 inflammatory environment in the gut that promotes the reemergence of disease upon cessation of therapy. Adjunct efforts that attempt to limit TH2 inflammation either by neutralizing effector TH2 molecules or by employing immunomodulatory bacterial therapies that promote Treg cell stability in the face of inflammation may be effective in increasing the rates of long-term tolerance acquisition in this disorder.

Patch-based immunotherapy has recently been employed as an alternative approach to OIT in inducing allergen-specific oral tolerance in FA. Tordesillas *et al*. used a non-oral desensitization route involving epicutaneous antigen delivery and showed that mice thus treated were protected from FA-related anaphylaxis ^124^. A population of gut-homing (tissue-specific) Treg cells that lacked Foxp3 protein but expressed latency-associated peptide was found to be selectively expanded, and these Treg cells did not suppress IgE production but inhibited mast cell activation. This report showed that skin-gut immune interaction is important for the induction of long-term oral tolerance in FA.

## Future directions

It is now clear that antigen-specific Treg cells play a key role in mediating peripheral tolerance to specific allergens in healthy individuals, but there are many questions left unanswered. First, the molecular mechanisms underlying the generation of pathogenic TH2 cell-skewed Treg cells in the periphery remain to be fully deciphered. Elucidating such mechanisms would enable therapeutic interventions that ‘reset’ the TH2 cell-like reprogramming of allergen-specific Treg cells to promote their tolerogenic functions ^68^. Such a reset may be further extended to cellular therapies in which allergen-specific Treg cells are expanded from the peripheral blood, modified away from the TH2 cell-like reprogramming and subsequently transferred back to patients. More broadly, the environmental factors associated with changes in lifestyle over the last decade may foster the loss of tolerance to allergens and need to be more specifically mapped. Particularly relevant is the role of altered microbial exposure early in life in shaping the Treg cell response ^101, 107^. A better understanding of how commensal microorganisms or their metabolites influence the generation of Treg cells and how dysbiosis may impact allergic responses would be highly informative in this regard. Thus, translating these advances to more effective manipulations of the allergen-specific Treg cell responses may offer novel therapeutic approaches in allergic disorders.
